# Improved therapeutic outcomes of thermal ablation on rat orthotopic liver allograft sarcoma models by radioiodinated hypericin induced necrosis targeted radiotherapy

**DOI:** 10.18632/oncotarget.9848

**Published:** 2016-06-06

**Authors:** Long Gao, Jian Zhang, Tengchuang Ma, Nan Yao, Meng Gao, Xin Shan, Yicheng Ni, Haibo Shao, Ke Xu

**Affiliations:** ^1^ Department of Radiology, The First Affiliated Hospital of China Medical University, Shenyang, China; ^2^ Laboratory of Translational Medicine, Jiangsu Provincial Academy of Traditional Chinese Medicine, Nanjing, China; ^3^ Department of Imaging & Pathology, Theragnostic Laboratory, University of Leuven, Leuven, Belgium

**Keywords:** radioiodinated hypericin, microwave ablation, sarcoma, orthotopic liver allograft

## Abstract

Residual tumor resulting in tumor recurrence after various anticancer therapies is an unmet challenge in current clinical oncology. This study aimed to investigate the hypothesis that radioiodinated hypericin (^131^I-Hyp) may inhibit residual tumor recurrence after microwave ablation (MWA) on rat orthotopic liver allograft sarcoma models.

Thirty Sprague-Dawley (SD) rats with hepatic tumors were divided into three groups: Group A received laparotomy MWA and sequential intravenous injection (i.v.) of ^131^I labelled hypericin (^131^I-Hyp) in a time interval of 24 h; Group B received only laparotomy MWA; Group C was a blank control. Tumor inhibitory effects were monitored with *in vivo* magnetic resonance imaging (MRI) and these findings were compared to histopathology data before (baseline, day 0) and 1, 4, and 8 days after MWA. In addition, biodistribution of ^131^I-Hyp was assessed with *in vivo* single-photon emission computed tomography-computed tomography (SPECT-CT) imaging, *in vitro* autoradiography, fluorescent microscopy, and gamma counting.

A fast clearance of ^131^I-Hyp and increasing deposit in necrotic tumors appeared over time, with a significantly higher radioactivity than other organs (0.9169 ± 1.1138 % ID/g, P < 0.01) on day 9. Tumor growth was significantly slowed down in group A compared to group B and C according to MRI images and corresponding tumor doubling time (12.13 ± 1.99, 4.09 ± 0.97, 3.36 ± 0.72 days respectively). The crescent tagerability of ^131^I-Hyp to necrosis was visualized consistently by autoradiography and fluorescence microscopy.

In conclusion, ^131^I-Hyp induced necrosis targeted radiotherapy improved therapeutic outcomes of MWA on rat orthotopic liver allograft sarcoma models.

## INTRODUCTION

Liver cancer treatment has advanced significantly [1–3]. Locoregional thermal ablations have evolved as crucial complementarily therapies [4–9] for liver cancer, and among these strategies, percutaneous microwave ablation (MWA) with real-time ultrasonographic guidance is favored because it is minimally invasive with fewer complications, and offers accurate targeting with substantially long-term curative effects compared to surgical resection [5, 10, 11]. Even with these superiorities, local or distant tumor metastases are identified after treatment, often due to large tumors volume and tumors that are adjacent to large vessels and/or vital organs which are continual challenges in efforts to completely eradicate disease. Reports regarding to the targeted treatment of residual tumors are also scarce.

Epstein's group [12] initially reported that injection of radioiodinated monoclonal antibody could target intra-cellular antigens exposed in necrotic and degenerating malignant tumor regions and that this internal irradiation could exploit antigen-antibody binding. ^131^I-chTNT (iodinated chimeric tumor necrosis therapy monoclonal antibody) has been applied to treat advanced lung cancer in China. However, clinical application of ^131^I-chTNT is limited by the macromolecule immunogenicity and bone marrow suppression.

Recently, Yun's group [13] used brachytherapy in mice bearing subcutaneous S180 tumors. With radioiodinated sennidin A (^131^I-SA), the authors targeted necrosis induced by injection of the vascular disrupting agent (VDA), combretastatin A4 phosphate (CA4P, i.v.), and noted tumoricidal effects and increased median survival in animals. However, that study was partly limited by the non-clinical availability of CA4P. In addition, the microenvironment of internal organs differed greatly from subcutaneous microenvironment for tumor growth.

Hypericin, a naturally occurring small molecule extracted from Hypericum perforatum [14], has been studied as a photosensitizer for photodynamic therapy [15]. Recently, hypericin has been afresh recognized due to its exceptional affinity for necrotic tissue [16–19]. Compared with some targeted drugs that just anchor to viable tumor tissues, hypericin could specifically and steadily bind to the tumor necrotic tissue with high target-to-nontarget ratios [16–19]. ^131^I-Hyp has also been identified as a potential therapeutic radiopharmaceutical in the field of so-called necrosis target radiotherapy compared with other previously identified specific agents [18, 19].

Furthermore, rather than hitting viable cancer cells undergoing numerous mutations that cause uncontrollable growth and escape from annihilation, targeting necrotic cells hold following three superiorities at least: (1) stable target with less heterogeneity; (2) generic target that widely exists in solid tumors; (3) superior specificity due to the exchange of macromolecules between intracellular and extracellular environment. Moreover, encouraging results have been published regarding the combination of VDA or thermal ablation with ^131^I-Hyp to treat subcutaneous solid tumors [13, 20, 21].

Based on the above considerations, we hypothesized that ^131^I-Hyp could specifically bind to the necrotic liver tumor induced by MWA and further irradiate the surrounding residual viable tumor with persistent cross-fire beta particles. To examine this, we established necrosis target spot by MVA, following intravenous injection of therapeutic nuclide ^131^I labeled hypericin (^131^I-Hyp) on rat orthotopic liver allograft sarcoma model. The targetability of ^131^I-Hyp was evaluated by SPECT-CT images, autoradiography, fluorescent microscopy and gamma counting. Tumor growth was dynamic monitoring by *in vivo* MRI and corresponding histopathology.

## RESULTS

### Animal models

**Figure 1:**
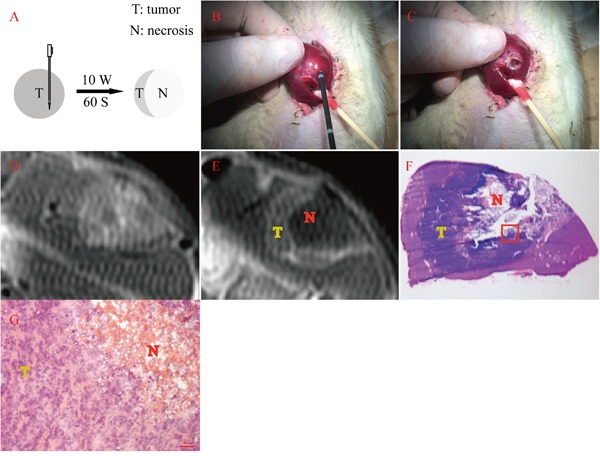
Residual tumor induced by MWA with its MRI and histopathology verification **A.** Schema chart of exocentric MWA. **B&C.** showed the tumor-bearing liver lobe before and after exocentric MWA. **D.** Contrast enhanced MRI before MWA showed a inhomogeneously elliptic enhancement on the edge of left liver lobe, with some hypointense signal in the center. **E.** Contrast enhanced MRI after MWA revealed a typical ring-enhancement at the same location, within which appeared extensive hypointense signal area. **F.** Gross photography of axial frozen section performed H&E staining corresponding to the same layer of Figure 2E. The collapse of the central area (N) and the adjacently dark blue area (T) represented necrosis and viable tumor respectively. **G.** This micrograph suggested the magnification field of the red rectangular frame delineated in Figure 2F, which showed both abnormal tumor cells and cracked necrosis incontrovertible (200 ×).

Rat orthotopic liver allograft sarcoma models were successfully established and residual tumors were induced with MWA (Figure 1A) as evidenced by contrast enhanced MRI (Figure 1D, 1E) and eventually post-mortem histopathology data (Figure 1F, 1G). All animals survived surgeries, tumor growth and *in vivo* imaging processes and only one animal died 2 days after the second laparotomy, probably due to severe abdominal distension and subsequent shock.

### Radiolabeling and *in vitro* stability studies

^131^I-Hyp labeling exceeded 90% according to ascending paper chromatography. ^131^I-Hyp was intact after 72 h by incubated in rat serum with radiochemical purity over 90%, which suggested a favorable stability.

### Biodistribution

**Figure 2:**
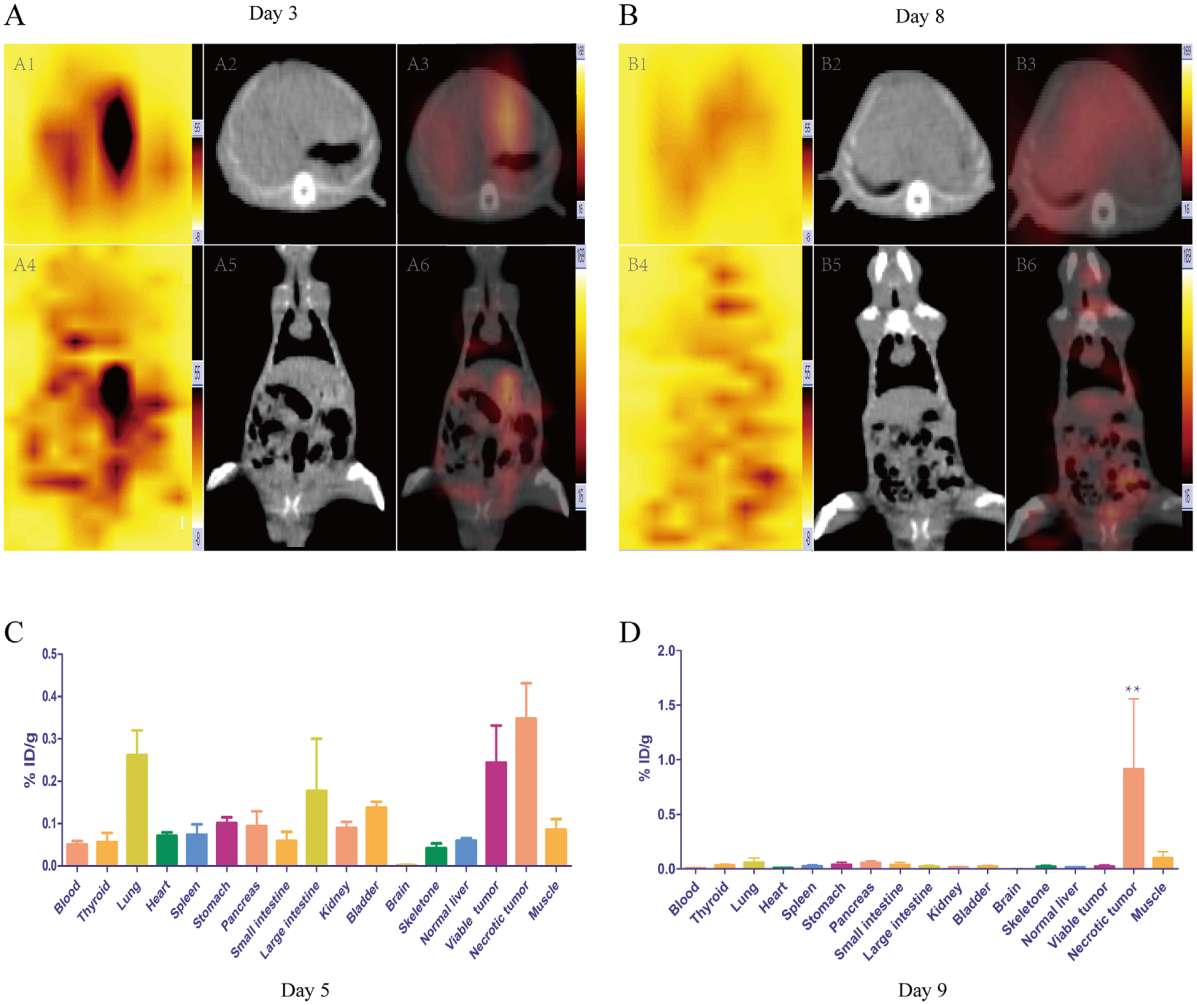
*In vivo* SPECT-CT **A, B.** and post mortem γ counting **C, D.** for rats receiving i.v. injection ^131^I-Hyp at different time point The planar axial scintigraphy (A1), CT (A2) and fusion images (A3) showed high radioactivity uptake in accurate location on day 3, while A4, A5 and A6 showed corresponding coronal planar expressions. B1-B6 showed analogous patterns for another rat on day 8. C&D represented the biodistribution of ^131^I-Hyp by mean of percentage of the injected dose per gram of tissues (% ID/g) on day 5 and day 9 respectively. ** The % ID/g was significantly higher than that of other organs (p < 0.01).

The biodistribution results were shown in Table 1. The lungs, large intestines, viable and necrotic tumors were radioactive compared to other organs on day 5 (Figure 2C). At last, increasing radioactivity in necrotic tumors on day 9 was significantly higher than that of other organs (0.9169 ± 1.1138, P < 0.01) (Figure 2D).

**Table 1:** Post mortem gamma counting results of following samples on day 5 and day 9 (4 d and 8 d post injection of ^131^I-Hyp)

Organ	% injected dose/g	
	Day 5	Day 9
Blood	0.0505 ± 0.0158	0.0086 ± 0.0074
Thyroid	0.0566 ± 0.0425	0.0338 ± 0.0186
Lung	0.2609 ± 0.1175	0.0588 ± 0.0698
Heart	0.0709 ± 0.0158	0.0105 ± 0.0061
Spleen	0.0739 ± 0.0478	0.0275 ± 0.0166
Stomach	0.1009 ± 0.0280	0.0399 ± 0.0375
Pancreas	0.0938 ± 0.0695	0.0545 ± 0.0282
Small intestine	0.0588 ± 0.0432	0.0372 ± 0.0385
Large intestine	0.1774 ± 0.2453	0.0244 ± 0.0155
Kidney	0.0892 ± 0.0306	0.0149 ± 0.0095
Bladder	0.1372 ± 0.0285	0.0242 ± 0.0144
Brian	0.0017 ± 0.0017	0.0013 ± 0.0010
Skeleton	0.0420 ± 0.0216	0.0228 ± 0.0178
Normal liver	0.0596 ± 0.0112	0.0146 ± 0.0077
Viable tumor	0.2431 ± 0.1762	0.0247 ± 0.0195
Necrotic tumor	0.3476 ± 0.1669	0.9169 ± 1.1138**
Muscle	0.0856 ± 0.0499	0.0982 ± 0.1015

The radioactivity was reported as a percentage of the injected dose per gram of tissues (% ID/g).

Numerical data were reported as the mean ± standard deviation.

** The injected dose per gram of necrotic tumor was significantly higher than that of other organs (p < 0.01).

### SPECT-CT imaging

On day 3, both axial and coronal fusion images depicted high radioactive nuclide concentration (“hot spots”) on left liver lobes at the tumor site. However, the signal was also very hyperintense in lung and intestinal canal (Figure 2A). Over time, radioactive intensity gradually weakened in hepatic lobes and increased in thyroids and intestinal canals especially in large intestines on day 8 (Figure 2B).

### MR imaging

**Figure 3:**
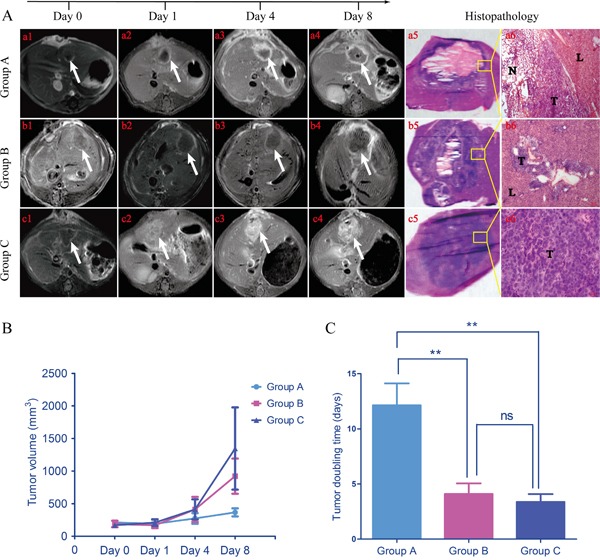
Representative *in vivo* MRI at different time points of each group and tumor growth trend During the 8 days following up, tumor grew comparatively slow in group A (a1-a4). While tumor growth seemed standstill for group B before day 4, the eventual volume still became far larger than baseline (b1-b4). For group C, tumor just kept growing rapidly with a gradually enlarging elliptic enhancement, which probably meant vigorous proliferation for tumor cells. a5, b5, c5. The macroscopic photographs of each group harvested from corresponding rats further confirmed the information deriving from *in vivo* MRI. a6, b6, c6. These photomicrographs denoted magnifying fields of yellow rectangular frames delineated in a5, b5 and c5 (50 ×, 100 ×, 400 × respectively). T, tumor; N, necrosis; L, liver. **B.** Tumor growth curves showed tumors in group B and C grew faster than that of group **A. C.** Tumor doubling time of group A was significantly lower than that of group B and C (A vs. B, p < 0.01; A vs. C, p < 0.01). There was no significant difference between group B and C (p > 0.05). **p < 0.01, ns p > 0.05.

At baseline (day 0), tumor volume appeared no significant statistical differences among three groups (211.1306 ± 40.0088, 188.2213 ± 50.0269, 169.8402 ± 16.3967 mm^3^; p>0.05). 24 hours after MVA, tumors showed obvious ring-enhancement in group A and B (Figure 3A, *a2, b2*), with a gradually increasing trend for group B. Over time, tumor in group A grew virtually slower than that of group B and C, which was certified by significantly prolonged tumor doubling time (A vs. B, 12.13 ± 1.99 vs 4.09 ± 0.97 days, p<0.01; A vs. C, 12.13 ± 1.99 vs 3.36 ± 0.72 days, p<0.01) and the eventually tumor volumes of three groups were 366.7713 ± 61.7467, 922.4149 ± 191.9072 and 1346.584 ± 630.6869 mm^3^ respectively. However, tumor doubling time showed no significant statistical difference between group B and C (4.09 ± 0.97 vs. 3.36 ± 0.72 days, p>0.05). Histopathology examinations (Figure 3A, *a5, a6, b5, b6, c5, c6*) further verified MR imaging findings. As shown in Figure 3A, *a5*, there was only a thin layer of tumor tissues, within which was a large area of necrosis. Although once suffered seriously deliberate damage, the residual tumor still maintained the exuberant vitality, rendering apparent tumor recurrence around central necrosis (Figure 3A, *b5*) and circumjacent blood vessels (Figure 3A, *b6*). There was no obvious necrosis in the tumor of group C (Figure 3A, *c5*) and these tumor cells revealed remarkable malformation as well as mitosis (Figure 3A, *c6*).

### Autoradiography

**Figure 4:**
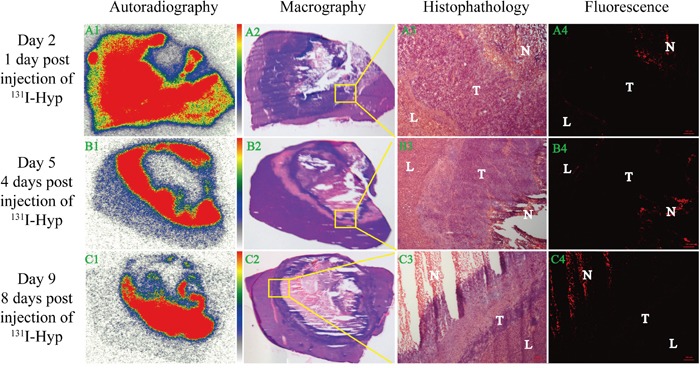
Dynamic *in vitro* autoradiography, fluorescence microscopy and corresponding histopathology results From the left to right column were orderly autoradiography (A1, B1, C1), macrography (A2, B2, C2), histopathology (A3, B3, C3) and fluorescence microscopy (A4, B4, C4) images, while from the top to bottom meant 1 day (A1, A2, A3, A4), 4 days (B1, B2, B3, B4) and 8 days (C1, C2, C3, C4) post injection of ^131^I-Hyp respectively. A3, B3, C3 (50 ×) denoted magnifying fields of yellow rectangular frames delineated in A2, B2, C2 respectively. A4, B4, C4 (50 ×) shared the same microscopic fields with A3, B3, C3. L, T and N denoted liver, tumor and necrosis respectively.

It was apparent that in the dead center of microwave ablation region, low uptake of ^131^I-Hyp appeared during the whole observation period according to autoradiography (Figure 4, *A1, B1, C1*) and macrography (Figure 4, *A2, B2, C2*) images. This might probably be due to that microwave energy destroyed the micro vessels that were the key transport corridors of drug delivery and more closed to the center, the vessels damaged more serious. High ^131^I-Hyp uptake appeared in tumor, normal liver and necrosis 1 day post injection of ^131^I-Hyp (Figure 4, *A1*), which suggested that the injection drug had not been sufficiently metabolized. Over time, radioactivity uptake in normal liver gradually weakened and high radioactivity uptake mainly concentrated in the border of viable tumor and necrosis (Figure 4, *B1*) 4 days post injection of ^131^I-Hyp. Meanwhile, the radioactivity ratio of this border over normal liver was 16.7 judging by optiquant software. On day 9 (8 days post injection), high radioactivity uptake still occurred at the border of viable tumor and necrosis with a enhancing trend of radioactivity but further weakening in central necrosis and normal liver (Figure 4, *C1*). The radioactivity radio of this border over normal liver reached 40.9. These results were basically identical with gamma counting (Figure 2C, 2D).

### Fluorescence microscopy and histopathology

Corresponding fluorescence images further verified this dynamic trend. 1 day post injection of ^131^I-Hyp, fluorescence intensity mainly focused on necrosis and lesser distributed in normal liver and tumor (Figure 4, *A4*). With time passed by, the fluorescence intensity can persistently concentrated in necrosis with a strengthening trend while barely not in normal liver and tumor (Figure 4, *B4, C4*).

## DISCUSSION

In this study, we established orthotopic liver allograft sarcoma model on rat and induced extensive tumor necrosis with MWA. ^131^I-Hyp could bind to necrotic tumor after MWA and inhibit residual tumor recurrence. According to the present results, we concluded that therapeutic outcomes of local thermal ablation can be improved on rat orthotopic liver allograft sarcoma models by ^131^I-Hyp induced necrosis targeted radiotherapy.

To the best of our knowledge, ideal specific tumor targeting tends to increase drug efficacy in targeted region and is less likely to damage collateral organs [27, 28]. Gamma counting results showed high non-targeted organs distributions of ^131^I-Hyp (lung, spleen, large intestine, bladder, etc.) at early time, and rapid clearances occurred within the half-life period of ^131^I (8.04 days). Eventually ^131^I-Hyp presented a persistent and increasing accumulation in necrotic tumor (Figure 2C, 2D), which guaranteed durable cross-fire ionizing radiation around the residual tumor. SPECT-CT images confirmed that prominent radioactive “hot spots” appeared at the position of the liver tumor. However, the signal was also very high in lung and intestinal canal (Figure 2A). This may attribute to the aggregation of hypericin which further activated immunizing phagocytosis conducted by organs rich in mononuclear phagocyte system (MPS) cells such as lung and spleen. Besides, hypericin was excreted via the hepatobiliary pathway to the intestinal canal and further concentrated in the abdomen on account of severe abdominal distension [29, 30].

Over time, the radioactive “hot spots” persistently appeared in the liver tumor. *In vitro* radioautography and fluorescent microscopy confirmed that ^131^I-Hyp persistently deposited in the target tissue (necrotic tumor) and rapidly excreted from non-target tissues (normal liver and viable tumor) (Figure 4). However, thyroid and intestine also showed high signals (Figure 2B). The deiodination of ^131^I-Hyp *in vivo* and serious flatulence caused by operation related adynamic ileus which could be certified by corresponding coronal CT image (Figure 2, *A5, B5*), may have accounted for this appearance. In addition, some excreta adhering on the fur of rat may also attribute to the high radioactivity in the abdominal cavity. Tumor inhibition was observed during the 8 day follow-up and ^131^I-Hyp could persistently concentrate in necrotic areas induced by MWA, significantly prolong TDT and inhibited recurrence of residual tumors compared to the control group. This was verified by *in vivo* MRI and corresponding histopathology (Figure 3). In terms of valid radiation dose, 50 Gy was essential to achieve tumoricidal effect for most neoplasms to our knowledge [33]. Ni's group [22] utilized ^131^I-Hyp (300 MBq/kg, i.v.) to treat R1-tumor bearing rats, estimated to correspond to a cumulative dose of about 5000 Gy over 8 days. In our study, ^131^I-Hyp was administered at 74 MBq/kg, which was according to previous reports [22, 33] estimated as approximately 1250 Gy during the same time. As a consequence, the theoretical tumor suppression could be achieved in our study.

**Figure 5:**
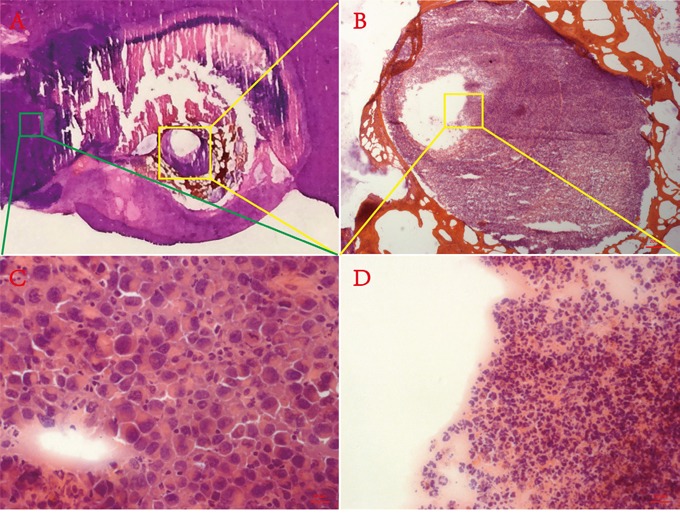
Ghost phenomenon **A.** Macrograph of H&E staining section containing necrosis, normal liver and viable tumor. **B.** Low-magnification microscopic field (50 ×) of yellow rectangular frame delineated in A. **C.** High-magnification microscopic field (400 ×) of green rectangular frame delineated in A. **D.** High-magnification microscopic field (400 ×) of yellow rectangular frame delineated in B.

Some unanticipated events occurred during the study. Figure 5 depicted the persistence of “tumor-like” tissue around the probe track (yellow rectangle), which could hardly be distinguished from normal tumor tissue using conventional H&E staining. Previous studies have indicated similar phenomena (“ghost phenomena” or “thermal fixation”) with VX2 tumor bearing rabbits and RIF-1 tumor bearing mice who received radiofrequency ablation (RFA) [31, 32]. To distinguish ablated and unablated tumor and normal liver tissue, Ni's group [32] used nicotinamide adenine dinucleotide (NAD) staining to confirm that this “tumor-like” tissue had no activity and was likely dead. They speculated that rapid increases in temperature of RFA denatured enzymes while preserving in tissue architecture and cytological detail. Similar to RFA, MWA had the same capacity to produce instantaneous high temperature with a more moderate process, therefore, this was likely a so-called “ghost phenomenon” or “thermal fixation”, and this represents the first report of such in W256 tumor bearing rats after MWA.

Whether therapeutic radionuclide can be delivered into the tumor microenvironment and persist there without causing adverse events is a key consideration of treatment. Compared with ^131^I-chTNT, ^131^I-Hyp remained in target tissue at greater concentrations with a ratio of retention time for 3 weeks to 3 days [34], largely due to the relatively smaller molecular weight (<1 kDa) of hypericin compared to macromolecular weight (>30 kDa) of monoclonal antibodies [33]. Thus, ^131^I-Hyp can more easily penetrate the tumor tissue microenvironment for the subsequent cross-fire radiotherapy.

There were some limitations in our study. First, the dose chosen for ^131^I-Hyp was based on published literature without experimental certification. Next, we failed to perform histochemical staining to verify ghost phenomenon in the process. Finally, we did not monitor survival, toxicity, or treatment-related side effects. In addition, the present research failed to illuminate the detailed mechanism of hypericin about its favorable affinity to necrosis.

### Conclusion

Our study did demonstrate that ^131^I-Hyp can persistently bind to necrotic tumor after MWA and further inhibit residual tumor recurrence on rat orthotopic liver allograft sarcoma models. MWA in combination with ^131^I-Hyp may offer synergistic anticancer therapy for tumor treatment and further studies on necrotic affinity mechanism of hypericin are warranted.

## MATERIALS AND METHODS

### Animal model

All experiments were approved by institutional Animal Care and Use Committee of China Medical University. Male Sprague Dawley rats (250-300 g) were provided by the Institutional Laboratory Animal Center. Animals were divided into donor (N=5) and recipient animals (N=30). For the donor animals, Walker 256 (W256) cells (2 × 106) were inoculated (percutaneously) in bilateral axilla every two weeks. Two weeks later, donor rats were anesthetized with 10% chloral hydrate (3 mL/kg, i.p.) and a standard laparotomy was performed to expose livers. Small tumor blocks (1 mm diameter) were harvested from donor rats. These tumor blocks were embedded in the left lobe of each liver of recipient rats that were anesthetized and laparotmized in the same way. Tumor embedding was accomplished using a handmade tumor conveyer. If necessary, a gelatin sponge was used for hemostasis. For all animals, layered sutures were used to close the abdominal cavity and recipient animal tumor blocks propagated until tumor diameters were 0.8 ± 0.2 cm. This was monitored every 3 days by MRI.

### Experimental design

**Figure 6:**
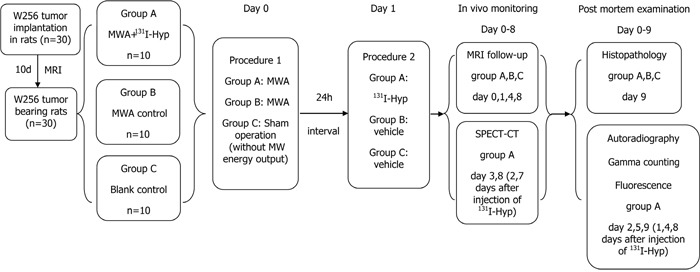
Flow chart of the experimental procedures in rats with orthotopic liver W256 allograft (MWA, microwave ablation; Hyp, hypericin; n, number).

Figure 6 depicts the experiment. Lugol's solution (1.2 g/L drinking water) was given to animals 3 days before treatment to prevent thyroid uptake dissociative ^131^I until the study end. Thirty rats orthotopic liver allograft tumors that were 0.8 ± 0.2 cm diameter were randomly divided into three groups: Group A (n = 10) received intra-operative MWA and injection of ^131^I-Hyp (74 MBq/kg, i.v.) after 24 h; Group B (n = 10) received intra-operative MWA and vehicle; Group C (n = 10) were blank controls treated with sham ablation and they received surgical interventions but without microwave energy delivery, and then they received vehicle after 24 h.

As ^131^I-Hyp alone has been documented to have little tumoricidal activity [18, 22], we deliberately did not include a control group of ^131^I-Hyp treatment alone. Also, we minimized the radiation injury during all procedures. Tumor inhibitory effects among all groups were monitored in real time using *in vivo* MRI prior to (baseline) and 24 h, 4 and 8 days after MWA application. Group A received *in vivo* SPECT-CT scans at days 3 and 8 to monitor distribution of radionuclides. To verify *in vivo* imaging findings postmortem, 3 rats from Group A were sacrificed at 24 h and 4 d and 8 d after ^131^I-Hyp treatment and then autoradiography, fluorescent microscopy, and gamma counting were performed. Rats of group B and C were histopathologically studied on day 9.

### Microwave ablation protocol

Rats with tumor diameters of 0.8 ± 0.2 cm were identified as MWA candidates. MWA was performed using a 2450 Hz cool-shaft (KY-2000; Kangyou Medical Instruments, Nanjing, China), transmitting microwaves via a 14-gauge cool-circle microwave ablation therapeutic probe (KY-2450B; Kangyou Medical Instruments, Nanjing, China) with an anterior pole of 5 mm, featuring a continuous sinusoidal microwave form. After anesthesia and standard laparotomy, liver tumors were exposed. Figure 1A and 1B indicated probe visualization at the tumor edge for retaining viable tumor material. Once the probe reached the designated location, microwave energy was delivered (10 W) for 60 s, and this was determined to be appropriate for inducing necrosis according to *in vitro* experiments on excised rat livers. After MWA of the tumor, the probe was gently removed with ongoing MWA emission until the probe tip approached the liver surface. This was intended to prevent tumor metastasis along the needle tract as far as possible (Figure 1C). The same abdominal closure was used on all animals as previously described. To confirm residual tumors, contrast enhanced MRI was performed 24 h before and after (Figure 1D, 1E) MWA procedures.

### Radiolabeling

Hypericin (purity 99%) was purchased from Purifa Co., Ltd, Chengdu, China. Sodium iodide (Na^131^I) with a specific activity of 740 MBq/mL (radionuclidic purity >99%) was purchased from HTA Co., Ltd, Beijing, China.

An Iodogen coating method was used to form ^131^I-Hyp. Hypericin was first dissolved in DMSO (0.5 mg/ml) and radio-iodination was triggered by adding the hypericin DMSO solution and Na^131^I solution onto an Iodogen-coated tube with (volume ratio hypericin/Na^131^I, 4:1). The mixture was allowed to incubate for 30 min at room temperature and the reaction was terminated by removal of the mixture. Radioactive labeling was confirmed using paper chromatography with filter paper (Whatman No. 1; GE healthcare, Piscataway, NJ) and 0.01 N HCL as a stationary and mobile phase. At last, the mixture was diluted with polyethylene glycol (PEG) 400 and propylene glycol (volume ratio, 1:1:1) and given to animals (74 MBq/kg i.v.) to study radiobiodistribution and tumor inhibitory effects.

### *In vitro* stability studies

To determine the *in vitro* stability of ^131^I-Hyp, the mixture of ^131^I-Hyp and rat serum (volume ratio, 1:9) was incubated at 37°C for 72 h. Plasma proteins were precipitated by adding 300 μL of ethanol and removed by centrifugation (12000 rpm, 10 min). The stability was assayed by using reversed-phase high performance liquid chromatography (RP-HPLC).

### Biodistribution studies

Rats (N = 3) from each time point (24 h, and 4 and 8 d post-injection of ^131^I-Hyp) were euthanized as depicted in the Methods. Then, brains, thyroids, lungs, hearts, spleens, stomachs, pancreas, small and large intestines, kidneys, bladders, skeletons, livers, viable and necrotic tumors, and muscle were sampled, weighed, and radioactivity was measured in each using an automatic gamma counter (2470 WIZARD PerkinElmer, city, MA). After corrections for physical decay and background radiation, activity was expressed as a percent of the injected dose/g tissue (% ID/g), which represented biodistribution of ^131^I-Hyp.

### SPECT-CT imaging

Group A received a SPECT-CT scan 2 and 7 days post-treatment of ^131^I-Hyp with a variable-angle dual detector SPECT with 16-slice CT (Symbia T; Siemens Medical Systems, Chicago, IL) unit. After anesthetization with chloral hydrate (0.3 g/kg, ip), rats were secured by the head on a platform in a supine position. SPECT-CT images were collected as follows: static image matrix size 128 × 128; acquisition count limit 50,000; SPECT tomographic image matrix 64 × 64; and continuous acquisition 15 s/frame × 24 frames.

### MR imaging

MRI was performed with a 1.5 T whole body MRI scanner (Echo speed; GE Co., New York, USA) with a rat coil (Chenguang Medical Technologies Co., Ltd, Shanghai, China). Rats were anesthetized using a small animal gas anesthesia machine (Matrix VMP; GENE&I, Beijing, China) with 2% isoflurane mixed with 20% oxygen/80% room air. Rats were then positioned supinely. Detailed scanning is described thusly:

T2 weighted image (T2WI): sequence pattern, fast spin-echo (FSE); repetition time (TR) / echo time (TE), 2920/88 msec; field of view (FOV), 100×100 mm; imaging acquisition matrix, 224×192; slice thickness, 2 mm; gap, 0.1 mm; total acquisition time, 3 minutes and 1 second.

T1 weighted image (T1WI): sequence pattern, spin-echo (SE); TR/TE, 550/24 msec; FOV, 100×100 mm; imaging acquisition matrix, 224×192; slice thickness, 2 mm; gap, 0.1 mm; flip angle, 80°; total acquisition time, 3 minutes and 5 seconds.

Contrast enhanced T1WI (CE-T1WI): sequence pattern, spin-echo (SE); TR/TE, 550/60 msec; FOV, 100×100 mm; imaging acquisition matrix, 224×192; slice thickness, 2 mm; gap, 0.1 mm; flip angle, 80°; Total acquisition time, 3 minutes and 31 seconds. CE-T1W images were acquired immediately after intravenous bolus of gadodiamide (GE Healthcare AS) at 0.2 mmol/kg [23].

### Processing and analysis of MR images

All MRI data were sent to an external dedicated workstation (Advantage Workstation, ADW 4.5, GE Medical Systems, New York, USA) and analyzed by three specialized radiologists. Regions of interest (ROI) which were tumor mass areas on each tumor-containing slice were manually delineated using a drawing tool. Tumor volume (TV) was calculated as follows: TV = tumor area on each tumor containing slice x (slice thickness + gap). Tumor doubling time (TDT) was calculated by TDT = (T - T0) ×log2/(logV - logV0), where (T - T0) indicates the time interval between two measurements, V and V0 denote the tumor volume at the two points of measurement [24].

### Autoradiography

Tumor-containing hepatic lobes of Group A rats were harvested as indicated and instantly frozen in OCT medium (Tissue-Tek; Sakura Finetek, Torrance, CA), and then serially sectioned (8- and 30 μm) using a cryotome (Shandon FSE, Thermo Fisher Scientific Co., Waltham, UK) and were thaw-mounted on glass slides. Then, 30-μm frozen sections were exposed for 12 h on a high-performance storage phosphor screen (Cyclone; Canberra-Packard, Ontario, Canada) to obtain autoradiographs. All acquired images were analyzed with Optiquant software (Canberra-Packard, Ontario, Canada) and radioactivity ratios of necrotic to normal hepatic tissue were estimated by manually indicating (drawing) ROIs. These 30-μm frozen sections then were stained with hematoxylin & eosin (H&E) and compared with autoradiographs.

### Fluorescence microscopy and histopathology

Next, 8-μm frozen sections were imaged under fluorescent microscopy with a digital camera (AxioCam HR, CarlZeiss, Germany) to observe hypericin fluorescence. After bright light and fluorescent microphotography, the 8-μm sections were stained with H&E and microphotography was again performed using the same fields of view as the previous fluorescent images. Group B and C rat tissues were treated the same to acquire microscopic images of tumor-containing liver lobes.

### Statistical analysis

Statistical analysis was conducted by using Graphpad Prism 5.0 software. Numerical data are reported as the mean ± standard deviation. One - way ANOVA was recruited to test differences among groups. Unpaired two-tailed Student t tests were performed for comparisons of two mean values. A P value of less than .05 was considered statistically significant.
